# Comparison of nutritional screening tools to assess nutritional risk and predict clinical outcomes in Mexican patients with digestive diseases

**DOI:** 10.1186/s12876-020-01214-1

**Published:** 2020-03-26

**Authors:** Mariana Chávez-Tostado, Gabino Cervantes-Guevara, Sandra Estefanía López-Alvarado, Gabino Cervantes-Pérez, Francisco José Barbosa-Camacho, Clotilde Fuentes-Orozco, Diana Mercedes Hernández-Corona, Tonatiuh González-Heredia, Guillermo Alonso Cervantes-Cardona, Alejandro González-Ojeda

**Affiliations:** 1 Departamento de Clínicas de la Reproducción Humana, Crecimiento y Desarrollo Infantil, Centro Universitario de Ciencias de la Salud, Universidad de Guadalajara, 44340 Guadalajara, Jalisco Mexico; 2 Department of Welfare and Sustainable Development, University Center of the North, University of Guadalajara, 46200 Colotlan, Jalisco Mexico; 3 Department of Biomedical Sciences, Health Sciences Division, Tonala University Campus, University of Guadalajara, 45425 Guadalajara, Jalisco Mexico; 4 Biomedical Research Unit 02, Western National Medical Center, Mexican Institute of Social Security, Avenida Belisario Domínguez # 1000, Col. Independencia, 44340 Guadalajara, Jalisco Mexico; 5 Department of Philosophical, Methodological and Instrumental Disciplines, Health Sciences University Center, University of Guadalajara, 44340 Guadalajara, Jalisco Mexico

**Keywords:** Length of stay, Morbidity, Mortality, Nutrition assessment, Nutritional index

## Abstract

**Background:**

The prevalence of malnutrition remains high in hospitals but no “gold standard” has been established to identify nutritional risks adequately. The Nutrition Risk Screening-2002 (NRS-2002), Subjective Global Assessment (SGA), and Controlling Nutritional Status Index (CONUT) are widely used screening tools, but their efficacy has not yet been compared in Mexican patients. Here, we aimed to compare the efficacy of these tools in identifying nutritional risks within the first 48 h of admission in a group of patients with gastrointestinal diseases.

**Methods:**

This was a cross-sectional study of 196 patients. The results of the screening tools, length of hospital stay, serum albumin and cholesterol concentrations, lymphocyte counts, age, body mass index (BMI), complications, and mortality were analyzed. Kappa (κ) statistics were applied to determine the degree of agreement between tools. The performances of the screening tools in predicting complications and mortality were assessed using binary logistic regression.

**Results:**

The NRS-2002, SGA, and CONUT tools identified nutritional risk in 67, 74, and 51% of the patients, respectively. The observed agreements between tools were: NRS2002/SGA, κ = 0.53; CONUT/NRS-2002, κ = 0.42; and SGA/CONUT, κ = 0.36. Within age groups, the best agreement was found in those aged 51–65 years (κ = 0.68). CONUT and length of stay were both predictive for the number of complications. The number of complications and serum cholesterol concentrations were predictive for mortality.

**Conclusions:**

The proportion of patients identified as having nutritional risk was high using all three screening tools. SGA, NRS-2002, and CONUT had similar capacities for screening risk, but the best agreement was observed between NRS-2002 and SGA. Only CONUT predicted complications, but none of these tools performed well in predicting mortality.

## Background

Undernutrition in hospitals and other health-care centers is currently a global problem, with a prevalence of 10–60% at hospital admission [1–5] It has been associated with clinical complications, increased morbidity and mortality rates leading to additional costs [2–4, 6, 7], increased length of hospital stay (LOS), increased frequency of hospitalization, and a decreased quality of life [1, 3, 8–10]. It often remains undetected because of the lack of awareness, knowledge, and clinical protocols to identify and treat this problem within hospitals [11, 12]. The risk of malnutrition tends to increase among patients in hospital gastrointestinal units because of alterations in food intake, impaired digestion, malabsorption, recurrent nausea, frequent fasting days, and vomiting [13]. Nutrition screening aims to detect the presence—and the risk—of developing undernutrition [13]. Thus, malnutrition needs to be assessed and identified before it can be addressed and resolved properly [1]. Numerous nutrition screening tools have been developed to detect the nutritional status of the patient [1, 13, 14] and should be assessed carefully within the first 24–48 h of admission to allow ample time for opportune treatments [15]; however, no consensus has yet been reached in terms of a “gold standard” screening tool [13, 14, 16].

The Subjective Global Assessment (SGA) tool is a simple and reliable screening instrument used to identify nutritional risk and is also the best predictor for hospital-related outcomes compared with other such indices. It was developed by Detsky et al. in 1987 [17], and is the most commonly used tool and includes information on a patient’s medical history (e.g., weight loss, dietary intake changes, gastrointestinal and functional impairments) and a physical examination (e.g., loss of subcutaneous fat, muscle wasting, ankle and sacral edema, and ascites). Each patient is finally classified as A (well nourished), B (moderately malnourished or suspected of being so), and C (severely malnourished). A limitation is that the SGA does not reflect subtle changes in nutritional status. Furthermore, it is subjective, does not account for biochemical values (e.g., visceral protein levels), and its sensitivity, precision, and reproducibility over time have not been extensively studied in some patient populations [17–20]. Nutritional Risk Screening (NRS)-2002 is one of the most used tools in hospitals worldwide. It was developed by Kondrup et al., and is meant to be a generic tool in a hospital setting [21]. It also identifies nutritional risk by investigating disease severity, weight loss, BMI, and food intake [21, 22], and also has demonstrated changes in important clinical outcomes, including mortality, in patients at risk of malnutrition [23]. Some reports have suggested that it is more suitable for evaluating nutritional risk and predicting clinical outcomes in hospitalized patients [1, 7, 10, 18], and has also been assessed and validated in hundreds of studies, including randomized controlled trials, and has been shown to be very reliable if administered by trained staff [20]. Nutritional risk is defined as a final score of ≥3 and no nutritional risk is defined as a score of < 3 [21, 22]. These two screening tools evaluate weight changes, altered food intake, and severity of disease to detect whether the patient’s nutritional condition is appropriate, or whether they have an increased nutritional risk.

The Controlling Nutritional Status (CONUT) tool is a relatively new index used to evaluate a patient’s nutritional status, and evaluation of its efficacy has begun [15]. It was first validated by Ulíbarri et al. in 2005 and identifies patients with different levels of nutritional status, depending on the final score: low undernutrition (0–4), moderate undernutrition (5–8), and severe undernutrition (9–12). It only takes into account the concentrations of serum albumin and TC, and total lymphocyte counts [6], so it is easier to use than other tools when applied by health-care providers [6, 15]. This screening tool is performed using these nutritional biochemical parameters because these measures are used as indicators of protein reserves [24] and nutritional status [25, 26].

The CONUT score also has some advantages over the NRS-2002 and SGA, such as simplicity and cost-effectiveness, but there are no reports comparing these three tools for screening among patients with gastrointestinal diseases. Therefore, the significance of the CONUT score is still unknown for these populations. There are only a few studies regarding undernutrition in patients with digestive diseases. In 2010, Filipović et al. [18], observed an undernutrition prevalence of 45.7% measured by the SGA and 63.9% by the Nutritional Risk Index (NRI) score in 299 hospitalized patients diagnosed with different digestive diseases upon admission. In another multicenter study, Amaral et al. [27] found that 36% of the patients were at risk of malnutrition according to NRS-2002 but only 9.7% showed such risk according to anthropometry. Tataoka et al. found that 38% of 40 hospitalized patients with Crohn’s disease were malnourished according to the SGA, but the NRS-2002 gave a result of 68%, and using the CONUT score, 25% of patients were considered severely malnourished (36). In a study by Allard et al., on 1015 patients of whom 30.4% had a gastrointestinal disease, they observed that 45% were malnourished according to the SGA [28]. One Mexican study by Alpízar et al. using the NRS-2002 found that 40.8% of hospitalized patients were malnourished [29]. Thus, the overall nutritional status of hospitalized patients has received increased attention from clinical professionals [16], with the aim of assessing and predicting clinical outcomes in patients with gastrointestinal diseases.

Here, we attempted to compare the differences in nutritional risk outcomes using SGA, NRS-2002, and CONUT in patients with different diseases and digestive disorders, and to estimate the degrees of concordance between these tools. We also aimed to evaluate the predictive ability of these systems in assessing morbidity and mortality according to clinical outcomes and nutritional scores in patients with gastrointestinal disorders.

## Methods

### Aims

We aimed to compare the efficacy of the NRS-2002, SGA, and CONUT scales in identifying nutritional risk within the first 48 h of admission in a group of patients with gastrointestinal diseases.

### Study design

This was a cross-sectional study of patients with gastrointestinal diseases during 2017 and 2018 in the gastrointestinal unit of the Civil Hospital of Guadalajara, “Fray Antonio Alcalde,” Mexico.

### Participants

The inclusion criteria for the study were: patients with gastrointestinal diseases; ages 18–90 years; at least 1 night of hospital stay with no surgery planned on the following morning. The exclusion criteria were: patients sent to the intensive care unit (ICU) during the first hours of admission or before screening assessment; patients with critical illnesses because of difficulty in follow-up and a possible lack of information; and pregnant or breast-feeding women, or those who had given birth within the past 6 months.

The sample size was decided based on the maximum number of patients who could be followed by staff adequately from the time of hospital admission until discharge or death. The initial sample was 202 patients, but six were excluded because of missing data. Thus, the final sample contained 196 patients. For convenience, all patients were divided into age groups of 15 years: ≤20, 21–35, 36–50, 51–65, and ≥ 66 years.

The blood samples were collected by venipuncture from the forearm of patients within the first hour of their admission. The blood collection was performed by the nursing staff of the gastrointestinal unit. All collection tubes were filled to the proper level, inverted 8–10 times, recorded, and placed in a − 80 °C freezer.

### Screening procedure

All nutritional risk screening was performed within 48 h of admission using the NRS-2002, SGA, and CONUT tools, and all tests were performed by the same dietitian. All anthropometric, clinical, and biochemical markers were also recorded. This included height, weight, body mass index (BMI), percentage of weight loss (in the previous 2 weeks and 6 months), age, gender, of the patient, clinical diagnosis, day of hospital admission, serum albumin and TC concentrations, and lymphocyte counts. All patients in our study were reclassified as “in nutritional risk” with a score of ≥3 by the NRS-2002, a score of B or C by SGA, and with moderate or severe undernutrition by CONUT.

### Clinical outcomes

The indicators for clinical outcomes were the incidence of complications, LOS, and mortality. Infectious complications included the appearance of any new infection, such as systemic inflammatory response syndrome, any abdominal infection, positive microorganism culture tests, pneumonia, urinary tract or catheter infections, pressure ulcers, abdominal fistula infection, fungal infection, and oral infections. Noninfectious complications included the appearance of anemia, upper and lower gastrointestinal tract bleeding, new onset of hepatic encephalopathy, myocardial infarction, or failure of any organ.

All information relating to the clinical evolution of the patient was documented in a medical file. The dietitian concerned followed up and collected all outcome indicators for patients until discharge, transfer, or death.

### Statistical analysis

Categorical variables are expressed as percentages and raw numbers, while continuous variables are expressed as the mean ± standard deviation. The analysis was performed using Student’s *t* test or the Mann–Whitney nonparametric *U* test for quantitative data, and the χ^2^ test or Fisher’s exact test for qualitative data. Differences were considered significant at *p* < 0.05. The prevalence of nutritional risk was calculated in the total population according to NRS-2002, SGA, and CONUT, and it was also calculated for specific age groups. Cohen’s kappa (κ) coefficient was calculated to measure the agreement between all screening tools for classifying nutrition risk with 95% confidence interval (CI) values. The Shrout classification [30] was used to interpret κ values as follows: 0–0.1, virtually no agreement; 0.11–0.4, slight agreement; 0.41–0.6, fair agreement; 0.61–0.8, moderate agreement; and 0.81–1, substantial agreement. For the binary logistic regression model, all anthropometric values, biochemical markers, clinical outcomes, and results of screening tools were included as independent variables to predict complications and mortality; *p* ≤ 0.2 was required for entry into the model. In the final model, *p* < 0.05 was considered statistically significant. Exponential (Exp) B values are reported as odds ratios (OR) with 95% CI values included in the final model or percentages. Model performance was assessed using the Hosmer–Lemeshow test to determine calibration across deciles of observed and predicted risk, and the accuracies of the NRS-2002, CONUT, and NRS-2002 tools were compared using *R*^2^ correlation coefficient calculations for each model. An analysis of the different tools’ sensitivity and specificity for quantitative variables was also conducted.

Statistical analyses were conducted using Excel 2007 (Microsoft, Redmond, WA, USA) and IBM SPSS Statistics software (version 20 for Windows; IBM Corp., Armonk, NY, USA). The statistical review in this study was performed by a biomedical statistician.

## Results

The study sample was comprised of 196 patients: 92 women (46.9%), and 104 men (53.1%). Ages ranged from 16 to 82 years; the mean was 46.4 ± 16.7 years. The mean BMI was 26.9 ± 7.6 kg/m^2^, and the mean LOS from admission to discharge was 5.5 ± 4.6 days. In terms of the age groups, 10 subjects were aged ≤20 years, 42 were aged 21–35 years, 64 were aged 36–50 years, 56 were aged 51–65 years, and 24 were aged ≥66 years. Twelve patients (6.1%) died during hospitalization, with no significant differences between the age groups. The most frequent gastrointestinal disease observed was acute pancreatitis in 70 patients (35.7%), chronic hepatic insufficiency in 36 (18.4%), peptic ulcer disease in 30 (15.3%), and extrahepatic biliary obstruction in 30 (15.3%). The remaining patients suffered different conditions such as inflammatory bowel disease, gastric neoplasms, caustic esophageal injury, liver abscesses, and other diseases, which together constituted 15.3% of the sample.

Within the groups of patients who did or did not die, statistically significant differences were found in LOS, number of complications, BMI, TC and serum albumin concentrations, and weight. Likewise, significant differences among patients with or without complications were observed in LOS, BMI, lymphocyte count, serum albumin concentration, weight, and in the percentage of weight loss in the previous 2 weeks. The clinical and demographic characteristics of patients are listed in Table 1.

**Table 1:** Clinical and demographic characteristics of patients

	Y/N	Total	Morbidity	Student’s ***t*** test ***p***-value	Mortality	Student’s ***t*** test ***p***-value
**Age (years)**	Yes	46.4 ± 16.7	48 ± 17.3	–	52.3 ± 15.1	–
	No		45.6 ± 16.5		46.1 ± 16.8	
**LOS (days)**	Yes	5.5 ± 4.6	9.1 ± 5.9	<0.001	9 ± 3.7	0.009
	No		3.6 ± 2.1		5.36 ± 4.6	
**NComp**	Yes	0.5 ± 0.8	–	–	2.5 ± 1.3	<0.001
	No				0.41 ± 0.68	
**BMI (kg/m**^**2**^**)**	Yes	26.9 ± 7.6	25.7 ± 4.9	0.001	25.05 ± 2.7	0.005
	No		28.1 ± 8.6		27.05 ± 7.9	
**Cholesterol (mg/dL)**	Yes	157.1 ± 128.2	137.4 ± 73.7	–	79.8 ± 30.8	0.031
	No		167.6 ± 148.4		162.1± 130.5	
**LCount (10**^**3**^**/ml)**	Yes	1586.08 ± 992.6	1377.1 ± 846.7	0.007	1188.3 ± 423.9	–
	No		1770.4 ± 1008.3		1663.06 ± 990.4	
**Albumin (mg/dL)**	Yes	3.3 ± 0.7	2.9 ± 0.8	<0.001	2.8 ± 0.5	0.023
	No		3.4 ± 0.68		3.3 ± 0.79	
**Weight (kg)**	Yes	71.2 ± 20.6	65.2 ± 13.8	0.001	67.5 ± 7.5	< 0.001
	No		74.3 ± 22.9		71.4 ± 21.2	
**% WL 6m**	Yes	7.4 ± 8.4	8.2 ± 9.3	–	8.2 ± 4.3	–
	No		7.01 ± 7.8		7.3 ± 8.6	
**% WL 2wk**	Yes	4.7 ± 4.6	5.8 ± 5.9	0.034	5.1 ± 3	–
	No		4.1 ± 3.7		4.7± 4.7	

*Abbreviations*: *BMI* body mass index, *LOS* length of hospital stay, *NComp* number of complications, *LCount* Lymphocyte count, *% WL 6m* percentage of weight loss in the previous 6 months, *% WL 2wk* percentage of weight loss in the previous 2 weeks

### Prevalence of nutritional risk

**Fig. 1:**
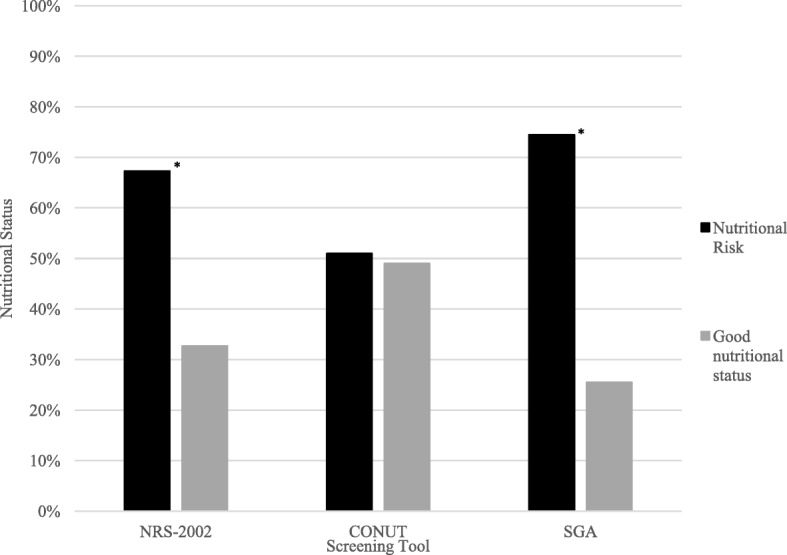
Nutritional risk of patients. Abbreviations: NRS-2002, nutritional risk screening 2002; CONUT, Controlling Nutritional Status Index; SGA, subjective global assessment. Note: * *p* < 0.001

**Fig. 2:**
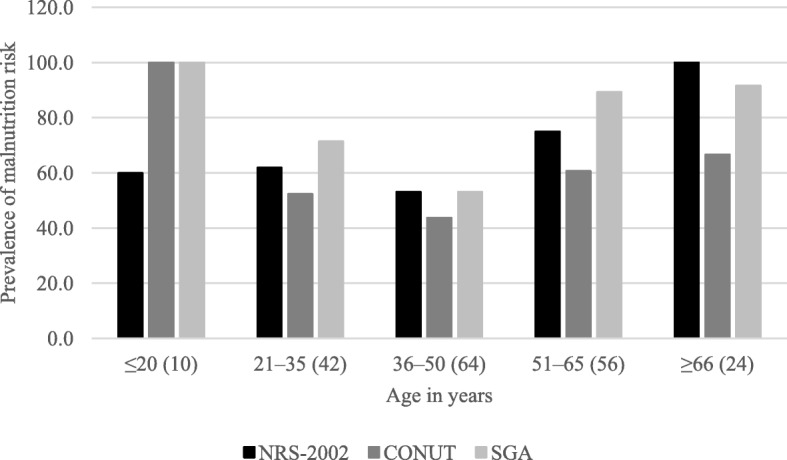
Prevalence of nutritional risk for different age groups. Abbreviations: NRS-2002, Nutrition Risk Screening 2002; CONUT, Controlling Nutritional Status Index; SGA, Subjective Global Assessment

The prevalence of nutritional risk among all subjects was 67% according to NRS-2002, 74% using SGA, and 51% with CONUT. Good nutritional status was observed in 32% of subjects according to NRS-2002, 25% with SGA, and 49% with CONUT. The differences between the three nutritional screening tools were statistically significant (*p* < 0.001). The results are shown in Fig. 1. For the different age groups, the highest prevalence of nutritional risk (100%) was observed in the age groups ≤20 and ≥ 66 years. The lowest nutritional risk was observed in the group aged 36–50 years (43.8%) according to CONUT. The results are presented in Fig. 2.

### Agreements between the screening tools

The results observed in the total population yielded a fair agreement between NRS-2002 and SGA (κ = 0.53), and between CONUT and NRS-2002 (κ = 0.42), but there was only a slight agreement between CONUT and SGA (κ = 0.36). When κ values were compared between age groups, the highest score found was between NRS-2002 and CONUT in the 51–65-year-old group, with moderate agreement (κ = 0.68). A fair agreement was observed between most of the remaining age groups. Nevertheless, there was no agreement in age groups ≤20 and ≥ 66 years with any of the screening tools, and only a slight agreement (κ = 0.3) between CONUT and SGA in the ≥66 years group. The results are presented in Table 2.

**Table 2:** Cohen’s κ coefficient between screening tools for different age groups

Age group (y)	*n*	NRS-2002/CONUT	NRS-2002/SGA	CONUT/SGA
≤20	10	0	0	0
21–35	42	0.22	0.57	0.41
36–50	64	0.44	0.49	0.44
51–65	56	0.68	0.52	0.31
≥66	24	0	0	0.3

Abbreviations: *NRS-2002* nutritional risk screening 2002, *CONUT* Controlling Nutritional Status Index, *SGA* Subjective Global Assessment

### Association of screening tools with complications and mortality

Regarding the presence of complications, 68 patients (34.7%) experienced some form, and of those patients, 56 (82.4%), 50 (73.5%), and 62 (91.2%) were detected to have increased nutritional risk by NRS-2002, CONUT, and SGA, respectively. Statistically significant differences were observed in association with all screening tools and the presence of complications (*p* = 0.001). The results are listed in Table 3. As for mortality, NRS-2002 and CONUT detected nutritional risk in 83.3% of the 12 patients who died and SGA detected 100%. The CONUT and SGA results had statistically significant associations with mortality, but NRS-2002 did not (Table 4). Death was observed in 10/12 patients (83.3%) with a normal BMI (18.5–24.9), and in 2/12 (16.7%) with BMI ≥ 25 kg/m^2^, but none with BMI ≤ 18.5 kg/m^2^. Complications were more frequent in 30 patients with a BMI ≥ 25 kg/m^2^ (44.1%), followed by 26 with a normal BMI (38.2%), and 12 with a BMI ≤ 18.5 kg/m^2^ (17.6%). These relationships were statistically significant for both mortality and the presence of complications (*p* = 0.002).

**Table 3:** Associations between screening tools and morbidity

		Without complications (*n* = 128)	With any complications (*n* = 68)	*p*	OR (95% CI)
NRS-2002	Absence of NR	40.6%	17.6%	**0.001**	3.2 (6.5–11.6)
	NR	59.4%	82.4%		
CONUT	Absence of NR	60.9%	26.5%	**0.001**	4.3 (2.3–8.3)
	NR	39.1%	73.5%		
SGA	Absence of NR	34.4%	8.8%	**0.001**	5.4 (2.1–13.5)
	NR	65.6%	91.2%		
Totals		65.3%	34.7%		

Values are shown as the odds ratio (OR) with 95% CI

Abbreviations: *NR* nutritional risk, *NRS-2002* Nutritional Risk Screening 2002, *CONUT* Controlling Nutritional Status Index, *SGA* Subjective Global Assessment

**Table 4:** Associations between screening tools and mortality

		Surviving patients (*n* = 184)	Dead patients (n = 12)	*p*	OR (95% CI)
NRS-2002	No NR	33.70%	17%	0.22	2.5 (0.54–11.9)
	NR	66.30%	83%		
CONUT	No NR	51.10%	17%	**0.021**	5.22 (1.11–24.4)
	NR	48.90%	83%		
SGA	No NR	27.20%	0%	**0.039**	1.09 (1.03–1.14)
	NR	72.80%	100%		
Totals		93.90%	6.10%		

Values are shown as the odds ratio (OR) with 95% confidence interval (CI)

Abbreviations: *NR* Nutritional risk, *NRS-2002* Nutritional risk screening 2002, *CONUT* Controlling Nutritional Status Index, *SGA* Subjective Global Assessment

The sensitivity and specificity of the screening tools regarding the presence of complications were as follows: NRS-2002, sensitivity 82.4% and specificity 40.6%; CONUT, sensitivity 73.5% and specificity 60.9%; SGA, sensitivity 91.2% and specificity 34.4%. Regarding mortality: NRS-2002 had a sensitivity of 83.4% and specificity 33.7%; CONUT, sensitivity 83.3% and specificity 51.1%; SGA sensitivity 100% and specificity 27.2%.

### Binary logistic regression analysis

By analyzing the relationship between nutritional risk and presence of complications using a regression model, an increase in each day of LOS was associated with a 63% increase in risk for the development of complications (*p* < 0.001, B = 0.49). Likewise, any one-step increases in malnutrition risk as measured by CONUT was associated with an increase of 3.98-fold in the rate of complications (*p* = 0.001, B = 3.98). This model explained the results with an accuracy of 54% with an *R*^2^ of 0.536. However, a regression model comparing nutritional risk and mortality showed that for each unit increase in TC concentration, the risk for mortality decreased by 5%; (*p* = 0.008) and for each increase in the number of complications, the mortality rate had an increase of 7.71-fold (*p* < 0.001). This model explained the results with an accuracy of 68% with an *R*^2^ coefficient of 0.679.

## Discussion

We conducted a study of nutritional risk as measured using the NRS-2002, SGA, and CONUT tools, as well as the association between the measurements and clinical outcomes such as LOS, complications, and death among inpatients with gastrointestinal diseases. An overall prevalence of nutritional risk detected with SGA was found in 74.5% of patients, 67.3% with NRS-2002, and in 51% with CONUT. By comparison, a study from Johns Hopkins Hospital reported a similar prevalence of 51.0%, with the highest risk being observed in the gastrointestinal department [31]. In our study, the nutritional risk was notably higher when measured using SGA, consistent with other studies, but ranges of 10–60% were observed in the upper level of risk [3, 5, 13, 32, 33]. A possible reason for this high prevalence might be that gastrointestinal diseases often lead to decreased nutritional status because of associated decreased food intake, and impaired digestive and absorptive functions [13]. Likewise, frequent fasting before planned surgery might also decrease nutritional status as shown by a Danish study, with a 57% rate of malnutrition in gastroenterological surgery departments [33]. Our findings are consistent with those of Filipović et al., who reported a 45.7% nutritional risk according to the SGA score in patients with gastrointestinal disease. They compared SGA, NRI, anthropometry, biochemical tests, and bioelectrical impedance analysis in patients with digestive diseases and found that the SGA tool was more sensitive in detecting predictor factors, although the sensitivity of NRI was also very high [18]. A similar study by Allard et al. [28] observed that of 318 patients with gastrointestinal disease, 45% were malnourished according to the SGA [34]. Our results are also similar to those of Tataoka et al., where 38% of 40 hospitalized patients with Crohn’s disease were malnourished according to the SGA, but with the NRS-2002 the incidence was 68%, and evaluation using CONUT found that 25% of patients could be considered as severely malnourished [35]. Thus, the CONUT tool seemed to underestimate the rate of undernutrition, as in our study. This might have arisen from the lack of basic nutritional markers such as recent loss of weight and appetite, or severity of disease.

Higher rates of nutritional risk were observed in our older patients (≥66 years), which agreed with previous studies [1, 13, 36], and in patients ≤20 years, as observed by Wang et al. [13]. However, the small number of individuals in these age groups might be a confounding factor so larger studies are needed to confirm this finding.

For all age groups, NRS-2002 and SGA detected more nutritional risk than did CONUT. This variability between screening tests could arise from the different scoring systems used. In SGA, the nutritional risk is observed more frequently in subjects with chronic undernutrition and its biggest disadvantage is the high interobserver variability [2]. However, when acute nutritional changes occur, they can be detected quickly using NRS-2002 and CONUT, but not with SGA, which might explain the discrepancies in our findings.

Consistent results were observed regarding the prevalence of undernutrition in hospitalized patients according to BMI. Thus, 9.2% of our patients were malnourished according to anthropometry, which is consistent with the findings of Ulíbarri et al. [6], who reported that 9.2% of patients were undernourished and with those of Amaral et al., who found that only 9.7% were malnourished—also according to anthropometry [27]. However, half of our population were overweight or obese with a mean BMI of 26.9 ± 7.6 kg/m^2^, similar to another study with a mean BMI of 25.6 ± 4.85 kg/m^2^ in hospitalized patients [6]. These results emphasize the important differences between actual BMI values and the nutritional risk of patients in identifying and predicting adverse clinical outcomes, given that BMI is a very subjective and unpredictable nutritional marker, while the nutritional risk scores could be associated directly with morbidity and mortality in our study. Here, patients with low BMI did not seem to have an increased risk of adverse clinical outcomes. In terms of mortality, 83% of the 12 patients who died had a normal BMI, and the remaining 17% of patients who died had a BMI ≥25 kg/m^2^, while none of the patients with a low BMI (≤18.5 kg/m^2^) died. Likewise, complications were more frequent in patients with a normal BMI (44.1%) and with BMI ≥25 kg/m^2^ (38.2%), than among those with a BMI ≤ 18.5 kg/m^2^ (17.6%).

### Agreement between nutrition screening tools

Moderate agreement was observed between NRS-2002 and SGA for the total population (κ = 0.53), which was nearly identical to previous findings by Wang [12] (κ = 0.51), and very similar to Kyle et al. [37], Velasco et al. [1], and Leandro-Merhi et al. [19], with κ statistics of 0.480, 0.620, and 0.46, respectively. These grades are very acceptable in routine clinical practice [6]. Some studies found that SGA predicted clinical outcomes well [32, 37] and presented the best agreement with the NRS-2002 in hospitalized patients [3]. Raslan et al. studied the association of malnutrition with clinical outcomes using NRS-2002 and SGA, and found that malnourished patients (defined by SGA) at nutritional risk (according to NRS-2002) were more likely to have the expected outcomes [32]. A Spanish study observed that NRS-2002 scores were associated with multiple complications, such as pneumonia, intestinal failure, fistulae, hyperglycemia, and death [19]. However, the CONUT scores had a fair agreement with NRS-2002 (κ = 0.42), but only a slight agreement with SGA (κ = 0.36). Surprisingly, when analyzing the agreement between age groups, moderate agreement was found between CONUT and NRS-2002 (κ = 0.68) in the 51–65-year-old age group. However, a poor agreement was found in age groups ≤20 and ≥ 66 years but these two groups both had small numbers of patients, which made the κ statistic very difficult to determine.

This study clearly showed that nutritional risk correlates with the incidence of possible in-hospital complications and with mortality in patients with gastrointestinal diseases, as observed in multiple previous studies emphasizing the importance of nutritional risk screening in the first hours of admission [1, 6, 13, 14]. In addition, lower levels of albumin, TC, and lymphocytes were observed in our group of patients at nutritional risk. This is also a known cause of higher numbers of complications and mortality as observed in our nutritional risk patients because previous reports have indicated that hypoalbuminemia and hypocholesterolemia were associated with increased short-term mortality, LOS, and complications [26, 38].

A potential bias in this study was in the selection of patients, which might have led to an underestimation of the prevalence of undernutrition in some. We excluded patients transferred to the ICU during the first few hours of admission because the information obtained during interviews could be limited. Furthermore, most biochemical markers were collected fully within the first 24 h of admission. However, this was done after the first day of hospitalization for some patients, which might have led to decreased levels of nutritional markers at the time of inclusion. Clearly, the high prevalence of nutritional risk observed in this study that was consistent with other papers [1, 13, 18, 27] demonstrates the importance of applying nutritional screening tests as a basic clinical element during the admission process and—more importantly—in patients with gastrointestinal diseases.

## Conclusions

The prevalence of risk of malnutrition was found to be high in patients with gastrointestinal disorders, which demonstrated the importance of including nutrition risk screening as an evaluation at admission. The NRS-2002, SGA, and CONUT tools worked well among patients with gastrointestinal diseases, but they performed differently for patients of different ages. Our findings suggest that NRS-2002, CONUT, and SGA can all be used to assess nutritional risk at admission in patients with gastrointestinal diseases, but NRS-2002 and CONUT are quicker screening methods than SGA that can be applied by an examiner with less training, and might serve as cost-effective screening methods for nutritional risk. In this sense, we encourage health-care professionals to use the CONUT nutritional screening tool to compare results with those found using SGA or NRS-2002. However, we recommend carrying out more studies with larger samples to establish whether this tool is valid and appropriate in all patients with gastrointestinal diseases.

## Data Availability

The data sets used and/or analyzed during the current study are available from the corresponding author on reasonable request.

## Abbreviations

NRS-2002: The Nutrition Risk Screening-2002
SGA: Subjective Global Assessment
CONUT: Controlling Nutritional Status Index
BMI: Body Mass Index
LOS: Length of Hospital Stay
TC: Total Cholesterol
NRI: Nutritional Risk Index
ICU: Intensive Care Unit
